# Socio-demographic, medical and social-cognitive correlates of physical activity behavior among older adults (45–70 years): a cross-sectional study

**DOI:** 10.1186/1471-2458-14-647

**Published:** 2014-06-25

**Authors:** Ilse Mesters, Stefanie Wahl, Hilde M Van Keulen

**Affiliations:** 1 School for Public Health and Primary Care (Caphri), Department of Epidemiology, Maastricht University, PO Box 616, Maastricht 6200 MD, The Netherlands; 2 TNO, Expertise Center Life Style, P.O. Box 2215, Leiden 2301 CE, The Netherlands

**Keywords:** Physical activity, Prevention, Cardiovascular disease, Intervention, Public health

## Abstract

**Background:**

Present study aimed to identify socio-demographic, medical and social-cognitive correlates of physical activity among Dutch older individuals.

**Methods:**

A systematic random sample of 2,568 Dutch participants aged 45–70 years filled out the validated modified Community Healthy Activities Model Program for Seniors (CHAMPS) questionnaire on physical activity. Socio-demographic and social-cognitive correlates were measured with validated instruments; medical correlates were checked by a general practitioner. The study had a cross-sectional design and the data collection ran from March 2005 until August 2006. Linear regression analyses were conducted to identify correlates of PA. We separated the findings for men from those for women to explore potential gender-specific associations.

**Results:**

Being female, living in North Limburg or North-Brabant, having a higher educational level, a higher perceived behavioral control, more knowledge about PA advantages, a stronger habitual PA behavior, having more action plans and a stronger intention to engage in PA were significantly associated with higher PA levels. Being older, being a smoker, having a higher body mass index (BMI), having a paid job, observing others being physically active and overestimating one's PA level were associated with being less physically active. Socio-demographic and medical correlates significantly explained 20% of the variance of PA behavior while social-cognitive correlates as attitude explained an additional 4% and intention together with actual control explained another 1% of the variance of PA behavior.

**Conclusion:**

There may be stable individual differences that influence PA in view of the fact that several socio-demographic and medical factors were not completely mediated by the socio-cognitive factors. The current study may help to focus PA interventions for individuals aged 45–70 years on influential socio-demographic, medical and social-cognitive correlates. Physical activity was significantly associated with age, gender, education, BMI, work situation, region of residence, smoking, awareness, advantages, descriptive norm, perceived behavioral control, habit, action plans and intention.

## Background

Physical Inactivity is considered to be an important risk factor for cardiovascular diseases (CVDs), which are the leading cause of morbidity and mortality worldwide [1]. Several reviews showed that physical activity (PA) may have a preventive effect on CVD mortality and morbidity [2-4]. Other benefits of PA are prevention of CVD risk factors such as diabetes type II, hypertension, anxiety and depression [5,6].

The Dutch PA guideline is based on international guidelines [5,7] and recommends adults to be physically active with a moderate intensity for 30 minutes 5 times a week. In 2011 59% of the Dutch population (18 years and older) and 70% of the people aged 55–75 years met the national PA guideline [8] For the development of an intervention to promote PA, knowledge about relevant and changeable determinants of PA is needed [9]. To understand how these determinants are mutually related, it has been recommended that determinant studies preceding behavioral change interventions should be grounded in theory [10]. Developers of health behavior interventions have often drawn on the Theory of Reasoned Action (TRA), respectively the Theory of Planned Behavior (TPB) [11], which explain the process through which people change their behavior [11,12]. According to these theories, the *intention state* for engaging in PA predicts future PA *behavior*[11]. The transition from intention to behavior is influenced by *actual control* (environmental factors, barriers, abilities, skills and action plans). The *intention state*, in turn, is determined by social-cognitive factors such as attitude, perceived norms and perceived behavioral control. *Attitude* refers to the evaluation of behavior in terms of cognitive and emotional advantages and disadvantages. *Perceived norms* include for example recognizing other individuals performing the type of behavior (*descriptive norms*). In this study, beliefs about whether specific social referents approve or disapprove engaging in PA (injunctive norms) was replaced by the construct *social support*, since the latter was described as having a much stronger influence on behavior [13]. *Perceived behavioral control* is the perceived ability to perform a health behavior. The social-cognitive factors are influenced by factors such as individual (e.g. personality, habit, and awareness), social-demographic (e.g. education, age) medical (e.g. having hypertension, diabetes, high BMI) and information factors (e.g. exposure to media). For instance, when people are in the habit of being physically active the predictive power of the social cognitive variables may be attenuated.

While several studies [14-16] have been conducted to investigate determinants of PA behavior, few [17,18] of them have focused on older adults (aged 45–70). However, several reasons exist to concentrate on older adults as target group for improving PA levels. Not only is 36.9% of the European population older than 50 years [19], but also this percentage will increase due to the aging of the population. Moreover, regular PA in older individuals contributes to an increase in longevity, a reduction of pain from arthritis, a decrease of risk to falls and fractures, and an increase in the ability to maintain functional independence [6]. Thus, stimulating PA among this large and growing group is of great relevance to minimize the public health burden.

The first aim of the study was to explore which socio-demographic, medical and social-cognitive factors have the strongest link with self-reported PA behavior in a population-based sample of adults aged 45–70 years living in the Netherlands.

Moreover, few Dutch studies have explored whether correlates of PA differ for middle aged men and women. It is expected that the associations may vary between the two [20].

Therefore, the second aim was to explore PA patterns and associated variables for men and women separately.

## Method

### Participants

A systematic random sample of patients was drawn from 23 general practices in the regions Limburg (19 practices) and North-Brabant (4 practices). These practices were connected to the RNH (Registration Network Family Practices) data base which allowed computer generated random selection of patients [21]. Selection variables included age (45–70 years), and ± 50% had general practitioner (GP) diagnosed hypertension based on the International Classification of Primary Care (ICPS code K86 or K87) [22,23] and about 50% had no hypertension. Additionally, selected persons should not be involved in other studies according to the general practice database and only one person per address was allowed to enter the study. Of 6,420 computer-selected participants 875 were excluded due to further selection criteria by the GP (e.g. unknown address, physically unable to comply to healthy lifestyle, not able to speak/read Dutch, life-threatening or malignant disorders). Invitations for participating in the study were mailed to 5,545 individuals and the 2,881 individuals who consented to participate received a printed questionnaire. Finally, 2,568 participants returned the printed questionnaire. For a full overview about the selection and enrollment of the Vitalum participants, see Van Keulen and colleagues [24]. The recruitment was done in waves and lasted from March 2005 until August 2006. The study was approved by the medical ethics committee of Maastricht University and Maastricht Medical Hospital (azM).

### Design

Baseline data of the Vitalum study were used for this cross-sectional study [25]. The Vitalum study simultaneously evaluated the efficacy of tailored print communication and telephone motivational interviewing, and their combined use for multiple health behaviors in individuals with and without hypertension and of diverse education levels. With the inclusion of participants with hypertension (systolic blood pressure > 140 mmHg), it was investigated whether the existence of a physician diagnosed medical CVD risk factor had an influence on PA behavior.

### Measurements

The TRA model indicates that other variables than intention and it antecedents (social-cognitive variables), can be associated with behavior. However, the influences of such background variables are expected to be indirect.

### Back ground variables

Age, gender, weight and height (for body mass index (BMI) calculation), highest completed education, work situation, marital status, family history of cardiovascular diseases [26], diabetes, living situation and native country were measured with a written questionnaire. Information about gender, hypertension status, and region of residence were provided by the GPs after participants agreed to take part in the study. The variable awareness was measured with two questions (for example Do you rate your PA level as low or high?; 1 = low, 5 = high [14] and the answers were compared to the reported PA levels in the questionnaire [26,27]. Accordingly, participants were categorized in two groups: 1. Overestimators who did not meet the guidelines but rated their PA level as high; 2. Underestimators who met the Dutch guideline but rated their PA level as low, or realists who estimated their PA level correctly. Habit was assessed by the frequency of engaging in physical activity (α .88) [15]; To what extent do you agree with the following statements: for example “Being physically active is something I regularly do” (1 = completely disagree, 5 = completely agree). The questionnaire further included one item for stress, two items for smoking behavior and two items for alcohol consumption. Stress was operationalized by asking whether the participants experience a lot of stress [28]. Smoking was assessed by asking the frequency and quantity of tobacco use [29]. Alcohol intake was measured by questions about the frequency and quantity of alcohol use, resulting in a drinking score below or above the national recommendation [30]. The alcohol national consumption guideline specifies a consumption of less than three glasses a day for men and less than two glasses a day for women [31]. As PA patterns may vary by season [32], the variable season was computed using the date on which participants returned the questionnaire.

### Physical activity behavior

PA was measured with the modified version of the Community Healthy Activities Model Program for Seniors (CHAMPS) questionnaire [33]. The original measure included 41 items [34], the modified questionnaire included 28 items about the frequency (times per week) and duration of physical activities (classified using six categories ranging from „less than 1 h · wk^-1^”, 1–2.5 h · wk^-1^, 3–4.5 h · wk^-1^, 5–6.5 h · wk^-1^, 7–8.5 h · wk^-1^ and “9 or more h · wk^-1^”). Included activities were walking leisurely/fast or briskly, cycling leisurely/fast or briskly, doing light/heavy gardening, doing light/heavy housekeeping, jogging or running, swimming, playing tennis or badminton, playing team sport indoors or outdoors, doing light exercises to maintain physical condition (stretching, flexibility training) and doing heavy exercises (fitness, strength training). Metabolic equivalents (METs) were determined for each activity on the basis of the compendium of PA by Ainsworth et al. [35].

MET levels were used as cut-offs to calculate the total number of weekly PA hours with at least a moderate intensity. Only activities with at least three METs counted as moderately intense activity for all participants [36]. Participants were classified as adhering to the PA guideline if they were physically active with at least moderate intensity for at least 2.5 hours a week. The number of weekly hours of at least moderate intensive activities was used as primary outcome.

The CHAMPS has been validated [33,37,38]. The reproducibility of the CHAMPS was shown to be good. e.g. [39].

### Social-cognitive factors

Questions about the following variables were included: attitude (advantages and disadvantages), perceived norms (descriptive norm and perceived social support), perceived behavioral control, habit strength, awareness, action plans and intention in form of stages of change (Transtheoretical Model) [40]. Factor analyses with principle axis factoring and promax rotation resulted in two underlying factors for attitude (advantages and disadvantages) and perceived norms (descriptive norm and social support). Habit and perceived behavioral control had one underlying factor. Sum scores calculated by summing up the items of the scale for the factors (advantages, disadvantages, perceived social support, descriptive norm, perceived behavioral control), were used in the data analysis. The social-cognitive concepts from the reasoned action model were operationalized for the current study according to the suggestions by diverse authors (Table 1).

**Table 1:** Description and operationalization of social-cognitive variables

**Concepts**	# **Items**	**Example of item question (answer option)**	**α**	**Reference**
Advantages *(attitude)*	13	Being physically active on at least 5 days a week for 30 minutes, improves my condition (1 = completely disagree; 5 = completely agree)	0.86	[16]
Disadvantages *(attitude)*	11	Being physically active on at least 5 days a week for 30 minutes, is very time consuming (1 = completely disagree; 5 = completely agree)	0.81	[16]
Social support *(perceived norm)*	5	Do important others (partner, family, friends, doctor or media) encourage you to be physically active according to the guidelines (1 = completely disagree, 5 = completely agree)	0.81	[16]
Descriptive norm *(perceived norm)*	3	Do important others (partner, family, friends) meet the physically activity norm (physically active for 30 minutes on at least 5 days a week) (1 = completely disagree, 5 = completely agree)	0.76	[16]
Perceived behavioral control	11	To what extent would you be able to be physically active on at least 5 days a week for 30 minutes, when you are tired? (1 = completely unable; 5 = completely able)	0.92	[17]
Action plan (*actual control)*	6	If you want to improve your PA level what would you do? “go for a brisk walk daily” (0 = no, 1 = yes)	-	[18]
Stages of change/intention	1	Which statement fits you best? Ranging from 1=”I have no plans to be physically active on at least 5 days for 30 minutes a day (no motivation) to 6=”I have been physically active on at least 5 days a week for 30 minutes for longer than 6 months” (maintainer).	-	[19]

### Statistical analyses

Statistical analyses were performed using SPSS 18.0. Statistical significance was assumed for *p-*values < .05. Descriptive statistics were performed to describe the sample. Information about missing values, outliers and data checking can be found elsewhere [24,25]. Cronbach’s alphas were used to evaluate the internal consistency of the social-cognitive scales. Hours per week moderately physically active acted as dependent variable and required square root transformation to achieve a normal distribution of the residuals. Linear regression was done to determine significant correlates of PA. For the linear regression (sequential multiple regression) the Enter method was used as it is a common procedure for model-based analyses e.g. [41].

To identify the unique contribution of background factors, social-cognitive factors, intention and actual control from the Theory of Reasoned Action, these factors were entered into the regression model in three steps. In the first step, background factors (e.g. age, hypertension, awareness) were entered followed by social-cognitive factors (attitude, perceived norms and perceived behavioral control) in step 2, and intention as well as action plans in step 3. During each step non-significant variables were manually deleted one by one and only significant variables stayed in the model. The same procedure was conducted for investigating gender-specific factors using select cases.

## Results

Of the 5,545 invited people 4,379 responded; 45% (n = 2,881) provided informed consent. Reasons for refusal included merely “no interest“or “no time”. The questionnaire was returned by 2,568 people (89% of the consenters). Questionnaires were checked at moment of reception for missing data and if present participants were contacted to complete the questionnaire. Sequential regression was employed to determine which background variables and social-cognitive factors were associated with self-reported PA behavior in older adults. Table 2 presents the background and social-cognitive factors of the sample.

**Table 2:** Socio-demographic characteristics, medical characteristics and social-cognitive factors of participants in the study (N = 2568)

**Variables**	**% or mean; SD; range**
**Background factors**	
Gender	
% Female	46.7
Age (mean; SD; range)	57.4; 7.1; 44 - 70
Native country	
% The Netherlands	99.6
Educational level*	
% Low	54.5
% Intermediate	23.4
% High	21.8
Living situation	
% Together	83.4
Work situation	
% Paid work	44.8
Marital status	
% Married/in a relationship	79.9
% Single/divorced/widowed	20.1
Region of residence	
% South Limburg	60.7
% North Limburg/North-Brabant	39.3
Hypertension	
% Hypertensive	51.9
Body mass index (mean; SD; range)	27.2; 4.7; 15.2 - 64.5
Diabetes	
% Diabetes	9.7
CVD family history	
% One family member with CVD	60.2
Perceived stress level	
% Less than normal	15.5
% Normal	51
% High	11.3
CVD family history	
% One family member with CVD	60.2
Smoking behavior	
% Smoker	21.1
Alcohol consumption**	
Glasses/day (mean; SD; range)	1.0; 1.4; 0 - 9
% Not meeting guidelines	14.0
Season	
% Spring	75.3
% Summer	9.0
% Autumn	2.7
% Winter	13.1
Physical activity	
CHAMPS: hours/week moderately physically active (mean; SD; range)	6.3; 4.9; 0 – 26.6
CHAMPS: % ≥ 2.5 hours/week moderately physically active	74.5
Gender specific	
Men (mean; SD; range)	5.8; 4.8; 0–26.5
Women (mean; SD; range)	6.8; 4.9; 0–26.6
**Social-cognitive factors**	
Awareness	
% Overestimator	60.0
% Underestimator/realists	39.6
Disadvantages (attitude)	38.5; 8.1; 1 - 55
Advantages (attitude)	48.5; 9; 11.6 - 65
Social support	14.1; 4.3; 3 - 25
Descriptive norm	9.7; 2.6; 3 - 15
Perceived behavioral control	38.6; 7.9; 11 - 55
Habit	11.2; 2.7; 3 - 15
Action plan	2.2; 1.1; 0 - 6
Intention (stages)	4.5; 1.9; 1 - 6

### Aim 1: Examining associated variables of PA

Table 3 shows the findings on physical activity as measured with the CHAMPS. Relatively prevalent activities, as walking leisurely and light housekeeping, were performed to some extent by about 80% of the respondents. For walking at a higher intensity this percentage was halved. Heavy housekeeping was included in the behavior of almost 67% of the respondents. Cycling leisurely seemed to be an option for 56% of the respondents and again this number was halved (28%) when the cycling intensity went up. Doing light or heavy gardening was mentioned by respectively 60% and 44% of the respondents. Only a few people reported to go jogging/running (9%), swimming (11%), playing (table) tennis/badminton (9%), and playing a team sport indoors or outdoors (6%).

**Table 3:** Champs data

			**How many hours on average**						
**How many times per week did you do underlying activities**	**MET**	**Amount per week**	**0**	**< 1**	**1-2.5**	**3-4.5**	**5-6.5**	**7-8.5**	**>9**
			**%**	**%**	**%**	**%**	**%**	**%**	**%**
		**M (sd)**	**n**	**n**	**n**	**n**	**n**	**n**	**n**
Walking leisurely	2.5	1.98 (2.49)	20.2	28.7	28.5	9.8	4.5	3.7	4.2
			518	737	732	252	115	96	109
Walking fast or briskly	4	.96 (1.82)	59.9	12.0	16.3	6.8	2.3	1.4	1.2
			1539	308	418	174	60	35	32
Cycling leisurely (<10 mph)	4	1.05(1.64)	43.8	23.4	21.5	7.4	2.1	.9	.8
			1125	600	551	191	54	24	21
Cycling fast or briskly (10-12 mph)	6	.70 (1.66)	72.4	8.3	9.9	5.3	2.0	.7	1.1
			1860	214	255	135	52	19	29
Doing light gardening	2.25	.89 (1.43)	39.9	30.8	22.1	4.1	1.0	.6	.8
			1024	790	568	106	26	16	21
Doing heavy gardening	4.4	.92 (1.73)	56.2	16	17.9	5.5	2.3	.6	1.4
			1444	412	459	140	60	15	37
Doing light housekeeping	2.5	3.17 (3.42)	21.5	15.4	24.1	11.6	6.6	5.1	14.6
			552	396	620	298	170	130	376
Doing heavy housekeeping	4.5	1.54 (2.13	33.1	22.0	27.1	9.9	3.2	1.8	2.6
			849	565	697	255	82	46	67
Jogging or running	7	.13 (.58)	91	4.3	3.6	.8	.1	.1	0.0
			2337	111	93	20	3	2	1
Swimming	7	.11 (.45)	88.8	7.4	3.4	.3	0,0	0,0	0.0
			2280	190	87	8	1	0	1
Playing (table) tennis, badminton	7	.17 (.69)	91.2	2.3	4.7	1.4	.3	.1	0.0
			2342	58	121	36	6	3	0
Playing a team sport indoors or outdoors	7.1	.11 (.51)	93.7	2	3.4	.7	.1	.1	0.0
			2406	52	87	18	2	2	0
Doing light exercise to maintain a physical condition, e.g. stretching or flexibility exercises	4	.33 (.79)	69.7	19.5	8.9	1.2	.3	.2	.1
			1790	501	229	30	7	4	2
Doing heavy exercises, e.g. aerobics, fitness or strength training	5	.27 (.84)	83.8	6.2	7.7	1.5	.7	.0	.1
			2152	159	197	39	17	1	2

In step one of the linear regression analysis, background factors significantly explained 20% of the variance of PA behavior (F(10, 2457) = 61.86, *p* < .000, R = .45, R^2^ = .20). When controlling for background factors, social-cognitive factors additionally explained 4% of the variance of PA behavior (F(13, 2397) = 57.48, *p* < .000, R = .49, R^2^ = .24, ΔR^2^ = .04). Furthermore, intention and actual control explained 1% of the variance of PA behavior in the third step, when controlling for the preceding factors. Age, gender, education, BMI, work situation, region of residence, smoking, awareness, advantages, descriptive norms, perceived behavioral control, habit, action plans and intention remained in the final model as significant correlates of PA behavior (see Table 4). In total, these factors explained 25% (F(15, 2390) = 53.45, *p* < .000, R = .50 ,R^2^ = .25, ΔR^2^ = .01) of the variance in PA.

**Table 4:** Linear regression of factors associated with physical activity

**Variables**	**Unstandardized coefficients**		**T**	**p-value**	**Confidence interval**	
	**β**	**SE**			**Lower**	**Upper**
Step 1 Background factors*						
Constant	2.166	.257	8.422	.000	1.662	2.670
Age	-.014	.003	-4.411	.000	-.021	-.008
Education level (low is reference category)						
DUMMY intermediate education	.138	.047	2.937	.003	.046	.229
DUMMY high education	.167	.048	3.460	.001	.072	.262
BMI	-.021	.004	-4.975	.000	-.030	-.013
Work situation	-.203	.047	-4.345	.000	-.294	-.111
Gender	.166	.039	4.219	.000	.089	.243
Region of residence	.210	.037	5.632	.000	.137	.283
Smoking behavior	-.187	.046	-4.052	.000	-.278	-.097
Awareness	-.233	.038	-6.176	.000	-.307	-.159
Habit	.136	.007	19.258	.000	.122	.150
Step 2 Social cognitive factors**						
Constant	1.086	.284	3.829	.000	.530	1.643
Age	-.010	.003	-3.156	.002	-.016	-.004
Education level (low is reference category)						
DUMMY intermediate education	.107	.046	2.303	.021	.016	.198
DUMMY high education	.144	.048	3.008	.003	.050	.237
BMI	-.019	.004	-4.474	.000	-.028	-.011
Work situation	-.182	.046	-3.941	.000	-.273	-.092
Gender	.195	.039	5.009	.000	.119	.271
Region of residence	.213	.037	5.773	.000	.141	.286
Smoking behavior	-.166	.046	-3.636	.000	-.256	-.076
Awareness	-.207	.037	-5.512	.000	-.280	-.133
Habit	.093	.009	10.934	.000	.076	.110
Advantages (attitude)	.007	.002	3.065	.002	.003	.012
Descriptive norm	-.016	.007	-2.163	.031	-.031	-.002
Perceived behavioral control	.027	.003	9.421	.000	.021	.032
Step 3 Intention and actual control*** FINAL MODEL						
Constant	1.250	.283	4.411	.000	.694	1.805
Age	-.011	.003	-3.470	.001	-.017	-.005
Education level (low is reference category)						
DUMMY intermediate education	.095	.046	2.052	.040	.004	.185
DUMMY high education	.135	.048	2.834	.005	.042	.228
BMI	-.019	.004	-4.408	.000	-.027	-.010
Work situation	-.183	.046	-3.969	.000	-.273	-.092
Gender	.191	.039	4.947	.000	.115	.267
Region of residence	.215	.037	5.849	.000	.143	.286
Smoking	-.165	.045	-3.644	.000	-.254	-.076
Awareness	-.211	.037	-5.660	.000	-.284	-.138
Habit	.083	.009	9.481	.000	.066	.100
Advantages (attitude)	.006	.002	2.531	.011	.001	.011
Descriptive norm	-.022	.007	-2.998	.003	-.037	-.008
Perceived behavioral control	.019	.003	6.038	.000	.013	.025
Action plan	.039	.018	2.248	.025	.005	.074
Intention (stages)	.077	.013	5.994	.000	.052	.102
Women**** FINAL MODEL						
Constant	1.935	.340	5.683	.000	1.267	2.603
Age	-.018	.004	-4.330	.000	-.026	-.010
BMI	-.016	.006	-2.822	.005	-.027	-.005
Work situation	-.126	.062	-2.028	.043	-.248	-.004
Region of residence	.257	.051	4.998	.000	.156	.358
Awareness	-.210	.052	-4.035	.000	-.313	-.108
Habit	.058	.012	4.643	.000	.012	.030
Perceived behavioral control	.021	.005	4.659	.000	.033	.082
Action plan	.058	.025	2.381	.017	.010	.106
Intention (stage)	.085	.019	4.571	.000	.048	.121
Men***** FINAL MODEL						
Constant	.946	.260	3.637	.000	.436	1.457
Education level (low is reference category)						
DUMMY intermediate education	.165	.061	2.726	.007	.046	.284
DUMMY high education	.217	.062	3.498	.000	.095	.338
BMI	-.024	.006	-3.784	.000	-.036	-.011
Work situation	-.128	.051	-2.501	.013	-.229	-.028
Alcohol consumption	-.171	.069	-2.486	.013	-.307	-.036
Region of residence	.174	.051	3.373	.001	.073	.275
Marital status	-.168	.069	-2.429	.015	-.304	-.032
Smoking	-.254	.063	-4.057	.000	-.377	-.131
Awareness	-.197	.052	-3.798	.000	-.299	-.095
Habit	.099	.012	8.431	.000	.076	.122
Perceived behavioral control	.019	.004	4.654	.000	.011	.028
Intention (stages)	.056	.017	3.266	.001	.022	.090

Dependent variable: Hours/week moderately physically active; *Step 1: R^2^ = .20, **Step 2: R^2^ = .24, ***Step 3: R^2^ = .25; ****Women: Step 1: R^2^ = .18, Step 2: R^2^ = .23, Step 3: R^2^ = .24; *****Men: Step 1: R^2^ = .22, Step 2: R^2^ = .25, Step 3: R^2^ = .25.

### In sum

#### Background factors

The results suggest that participants who overestimated their PA level, who were older, who smoked, who had a higher BMI and had paid work were less physically active. In addition, participants with stronger habitual PA behavior, with higher education levels, who were female, and who lived in North Limburg or North-Brabant had higher PA levels.

#### Social-cognitive factors

The results indicate that participants who perceived more advantages and had high perceived behavioral control were more physically active. Participants who perceived physically active role models were less physically active.

#### Intention

Participants, who formed more action plans and who had a stronger intention were more physically active.

### Aim 2: Examining whether the variables associated with PA vary by gender

#### Women

Among women, background factors explained 18% (F(6, 1133) = 41.58, *p* < .000, R = .425, R^2^ = .18) of the variance of PA behavior. Social-cognitive factors additionally explained 5% of the variance in PA behavior in step 2 (F(9, 1063) = 35.53, *p*<,000, R = .48, R^2^ = .23, ΔR^2^ = .05), and intention and actual control accounted for an additional 1% of the explained variance in PA behavior in step 3 (F(9, 1104) = 38.67, *p* < .000, R = .49, R^2^ = .24, ΔR^2^ = .01).

#### Men

Among men background factors explained 22% (F(10, 1310) = 36.75, *p* < .000, R = .47, R^2^ = .22) of the variance of PA behavior. Social-cognitive factors additionally explained 3% of variance in PA behavior in step 2 (F(11, 1292) = 38.73, *p*<,000, R = .50, R^2^ = .25, ΔR^2^ = .03). Intention and actual control did not explain additional variance in PA behavior in step 3 (F(12, 1287) = 36.36, *p* < .000, R = .50, R^2^ = .25, ΔR^2^ = .00).

The following factors varied by gender: Women who were older were less physically active. In contrast, women who made more action plans were more physically active. Men with a higher education level were more physically active. On the contrary, men who drank alcohol above the norm, who smoked, and who were married or living together, were less physically active. For a full overview of factors see Table 4.

## Discussion

This study was one of the first to investigate socio-demographic, medical and social-cognitive factors associated with PA behavior in large sample of individuals aged 45–70 years in the Netherlands. The present study showed that most popular PA activities among older adults appeared to be walking and cycling leisurely and household activities. According to the CHAMPS three-fourths of the respondents reported to perform at least two and a half hours of moderate physical activity a week. Previously we have shown that multiple item instrument as the Champs may overestimate PA level [42]. Therefore, it should be mentioned that the actual percentage might be lower. In this study 25% of the variance in PA was explained by age, gender, education, BMI, work situation, region of residence, smoking, awareness, advantages, descriptive norm, perceived behavioral control, habit, action plans and intention. Therefore, these variables were considered to be important to target in interventions aimed to increase PA in older adults. The later intervention study addressing these elements appeared successful in improving PA [25]. Although comparable studies on PA behavior measured with the CHAMPS questionnaire were not found, the present findings corroborated those of previous Theory of Reasoned action (TRA)/Theory of Planned Behavior (TPB) studies on physical activity regarding relevant correlates and percentages of variance explained. In a meta-analytic review of 79 studies, Hagger et al. reported that the TRA accounted for 26% of the variance in PA behavior and the TPB explained 27% of the variance in PA behavior [12]. Similar percentages were also found in PA studies among older adults [43,44].

Several of our background variables were not completely mediated by the TRA/TPB constructs indicating that there may be stable individual differences that influence PA. The shown influence of background factors may enrich our understanding of PA behaviour.

Even though many correlates were identified in the current study, 75% of PA variance remained unexplained. As stated, our percentages were similar to findings of other studies using diverse behavioral outcome measures, but a lack of correspondence between the social–cognitive measures in which we defined the investigated behavior as being moderately physically active for at least 30 minutes on five or more days a week and the CHAMPS measure (mapping specific behaviors) cannot be excluded [11].

To increase the explained variance, several predictors have been suggested as additions to the model as the contribution of environmental factors, diverse health status indicators, perceived health, (perceived) lack of leisure time, mood disturbance and perceived PA effort [45]. In this study three health status indicators, diabetes, hypertension and BMI were added to the predictor list. Only BMI seemed to have an effect on PA. The observed negative influence of BMI on PA levels was in line with previous studies among the same age group [18,43]. Gauvin et al. noted that women with higher BMIs experience PA not as pleasurable and feel embarrassment when seen in public with exercise clothes [43]. However, this explanation did not hold for men and requires more research. In future interventions this health indicator should get extra attention.

The decline in PA levels with age is consistent with other studies conducted among a similar age group [18,46]. Norman et al. stated that poorer health or perceived lack of good health may induce that older people engage less often in PA [18]. Dergance et al. believed that fear of injuries may contribute to the decline in PA level among older individuals [47]. Additionally, Berger et al. suggested that unfavorable cultural expectations and norms exist about PA among older adults [46]. The general perception is that social norms demand that people relax and that more vigorous PA is not an appropriate behavior for older adults [43]. Unfortunately, social norm was not measured in the current study to support this assumption since it rarely was a significant predictor in previous studies on PA [44]. Another reason for the decline in PA levels by age is given by Norman and colleagues, who claimed that the lower PA level of aging people was associated with a lower level of occupation and occupational activities among older adults [18].

In contrast to other studies among the same age group [18,46], the current findings indicate that women older than 45 years were more physically active than men of same age. This was also found in a Scottish sample among individuals aged 75 or older [48]. The increased activity among older women could be explained due to extra available time caused by a decrease in workload at home and care-giving for children, who live now outside the home [49]. Another reason could be that men achieve their PA levels by playing sports (e.g. football), but with increasing age these activities become harder to continue [50].

Besides the current study, several researchers found that employees older than 45 years are less physically active e,g, [18]. One explanation is that employees may be physically active at their work place or a have a physically active job, an aspect that is often not taken into account in PA questionnaires. Moreover, Berger at al. observed that the more physically demanding work is, the less likely people are to engage in PA outside work, meaning PA at work compensates PA in leisure times [46]. However, the current study cannot support this argument as PA at work was not measured.

Consistent with the findings reported elsewhere [18,48], the current results confirmed that high educated older individuals have higher PA levels. An explanation is that high educated individuals have a better health consciousness and more knowledge about advantages than lower educated individuals. There are also indications that high educated individuals have a higher perceived behavioral control and perceive fewer barriers engaging in PA [51]. In the current study, a t-test analysis was conducted comparing high versus low educated, which partly supported the assumptions. Compared to low educated, perceived behavioral control was higher among high educated but non-significantly. In addition, high educated individuals experienced significantly fewer disadvantages (barriers) but also fewer advantages compared to low educated.

Smokers appeared less physically active than non-smokers. This was also found by others [18,46]. The finding can be explained by the fact that health risk behaviors cluster, and that smoking has a disruptive effect (e.g. decreased lung function) on PA performance [52].

Additionally, the results of this study suggest that men are less physically active when drinking alcohol above the national norm. The few studies that investigated the relationship between alcohol consumption and PA found no significant association [50,53]. To our knowledge no study exists regarding the influence of alcohol on PA behavior among older adults and possible gender differences.

The present data, and those of others [54], identified individuals living in North Limburg and North-Brabant as being more physically active than individuals in South Limburg. Mulder reported that South Limburg with 46.8% has the lowest number of individuals older than 12 years meeting the national physical activity norm [54]. South Limburg, in contrast to the North, is a hilly area which might cause the lower level of PA. Several studies considered hilly terrains as barrier for PA [55].

With respect to the social-cognitive factors, the current study has demonstrated that individuals, who were not aware of and overestimated their own PA levels, were less physically active. Other studies conducted among Dutch adults (18 and older) confirmed this finding [56,57]. Ronda et al. concluded that 61.1% of the individuals with inadequate PA levels overestimate their PA level and thus making the motivation to increase PA in these groups difficult [40].

The positive influence of perceived behavioral control on PA found in the present study is in line with previous studies among the similar age group [58,59]. Perceived behavioral control is acknowledged as the most important psychological factor for PA e.g. [12,45]. High perceived behavioral control allows people to set reachable goals and overcome barriers, which enable them to be physically active [60]. Rovniak et al. [61] found in university students that perceived behavioral control was mediated by self-regulation strategies such as goal setting, self-monitoring, planning and problem solving. Moreover, personal achievement to be physically active, experiencing other people accomplishing engagement in PA (social comparison) or verbal persuasion by others to participate in PA increase perceived behavioral control [63]. In the current study, individuals observing other persons being physically active or having social support also had significantly higher perceived behavioral control, as a t-test assessment revealed.

Several studies demonstrated that action plans are important to overcome the gap between intention and actual behavior [62-64]. This means, specifying PA behavior using parameters such as ‘when’, ‘where’ and ‘how’, leads to actual PA behavior. According to Reuter et al., older adults are more successful in implementing their plans despite barriers due to more experienced self-regulation mechanisms (e.g. goal-setting) [62]. However, our study did not confirm that adults who are older have more action plans.

Consistent with the findings reported in other studies among broader age groups [e.g. 12], the present study showed that habit strength was associated with PA behavior. Behaviors have been once initiated by rational choices and later they have been formed to habits, which are triggered by cues without cognitive processes [65]. Additionally, positive experiences (e.g. emotions and enjoyment) of PA are important for the formation of habits [65]. Positive feelings during PA increase positive attitude [65]. Keviniemi et al. found that positive affective associations can serve as cognitive shortcuts to PA [66]. This means that individuals decide to engage in PA without using a rational decision-making process each time, but because they expect, for example, enjoyment from the PA. In line with other studies [67], the finding of the present study also support that individuals believing more strongly in the health benefits of PA are more physically active.

In the current study, participants were not motivated to engage in PA by observing other physically active people in their environment. This is in contrast to most studies among similar age groups [67,68]. However, Wilcox and colleagues studied sedentary behavior among women 40 years and older. They also found that women who see others exercise, have a more sedentary behavior [69]. Moreover, a study among women 20 to 50 years old demonstrated that female urban Latinas and urban African Americans knowing people who exercise were less physically active [70]. Unfortunately, neither study supported their finding with an explanation. Reasons might be that they do not perceive others as role models for PA so that they cannot identify themselves with others.

Consistent with the findings reported elsewhere [17,71], older individuals with the intention to take part in PA are also more physically active. According to Fishbein & Ajzen, forming the intention to engage in PA is translated into actual PA behavior [11].

### Study strength and limitations

The current study had several strengths including the origin of the study population, the large sample size, the random selection from different GP practices, the inclusion of medical characteristics (e.g. hypertension), and the generalizability of results with regard to gender and education level. Moreover, it is one of the first studies concentrating on factors influencing PA in older individuals.

Limitations of the study are the cross-sectional design and the measurement of PA behavior through a self-reported questionnaire. Self-report measurements require from participants good memories and estimation skills. Consequently, measurement errors may exist due to social desirable answers or lack of valid recall [72]. Moreover, the PA measure lacks detail and specificity because it focuses on common activities and misses workplace physical activity. Thus, PA behavior may be over- or underestimated [73]. Another limitation of the study is the use of a long questionnaire to measure PA and the other variables [74], which may result in nonresponse and invalid results [75]. In the Netherlands about one third of the population (aged 30–70 years) suffers from hypertension [76]. For research purposes the number of people who have hypertension was higher (50%) than normal. Nevertheless, hypertensive people did not seem to be more physically active than normotensive people in this study. Finally, the study was limited due to the lack of relatively more objective measures such as activity monitors to validate the self-report questionnaires, because the use of these objective measures was considered too time-consuming and expensive in such a large population [77].

## Conclusion

The current study adds to the literature by identifying important socio-demographic, medical and social-cognitive associates of PA in individuals aged 45–70 years. Consequently, it contributes to the ability to develop PA interventions tailored to older adults. The following correlates of PA should be considered in the development of these interventions: age, gender, education, BMI, work situation, region of residence, smoking, awareness, advantages (attitude), descriptive norm, perceived behavioral control, habit, action plans and intention. By considering these factors in regional or national PA interventions, a higher level of PA among older adults could be stimulated and as a result the public health burden may be minimized through a decrease in CVDs among this large population group.

## Abbreviations

BMI: Body mass index; CVDs: Cardiovascular diseases; CHAMPS: Community healthy activities model program for seniors; PA: Physical activity; TPB: Theory of planned behavior; TRA: Theory of reasoned action.

## Competing interest

The authors declare that they have no competing interests.

## Authors’ contribution

HMVK carried out the Vitalum Study, helped with the statistical analysis, and revised the manuscript critically. IM helped to draft the manuscript and to interpret the data, and revised it critically. SW performed the statistical analysis, did the interpretation of the data and drafted the manuscript. All authors have seen and approved of the version to be published.

## Pre-publication history

The pre-publication history for this paper can be accessed here:

http://www.biomedcentral.com/1471-2458/14/647/prepub
